# Radioactive iodine effects on ovarian reserve: a systematic review and metaanalysis

**DOI:** 10.3389/fendo.2026.1636824

**Published:** 2026-03-10

**Authors:** Salwa Q. Bukhari, Hyder Mirghani

**Affiliations:** University of Tabuk, Tabuk, Saudi Arabia

**Keywords:** anti-Müllerian hormone (AMH), differentiated thyroid cancer, follicle-stimulating hormone (FSH), ovarian reserve, radioactive iodine RAI (I-131)

## Abstract

**Background:**

Differentiated thyroid carcinoma (DTC) is common, and its rate is on the rise globally. Radioactive iodine, RAI (I-131), is widely used as an adjuvant therapy or for remnant ablation. There is growing awareness about the effects of RAI (I-131) on ovarian reserve.

**Materials and methods:**

This meta-analysis aimed to evaluate whether RAI therapy impairs ovarian reserve as assessed by anti-Müllerian hormone (AMH) and follicle-stimulating hormone (FSH) levels. A systematic literature search was conducted in PubMed, MEDLINE, Google Scholar, and EBSCO for relevant articles published in the English language. The literature search was conducted during October and November 2024, and the studies were included if they were published in the period from 2015 to 2024. The keywords used were ovarian reserve, ovarian function, female fertility, radioactive iodine, RAI (I-131), follicle-stimulating hormone (FSH), and anti-Müllerian hormone (AMH). MeSH terms used: Iodine Radioisotopes, Ovarian Reserve, and Thyroid Neoplasms.

**Result:**

Out of the two hundred and sixty-nine studies retrieved, 160 remained after duplication removal, of which 17 full texts were screened. However, only six studies (with 430 patients) were included in the final meta-analysis. AMH level were significantly lower after RAI (I-131) with a mean difference (MD) of 1.96. 95% *CI*, 0.53 to3.40. The levels were higher in the RAI (I-131) group after removing studies with significant heterogeneity, MD, 0.36. 95% *CI*, 0.17 to 0.55. FSH levels were significantly higher following RAI (I-131), MD -1.07, 95% *CI*, -2.02 to-0.13.

**Conclusion:**

Current evidence shows a significant reduction in anti-Müllerian hormone (AMH), and significant increase in FSH levels after RAI (I-131), but data are heterogeneous. Larger prospective studies with standardized outcome measures are needed.

## Introduction

Differentiated thyroid carcinoma is common, and radioactive iodine RAI (I-131) therapy is used for ablation of thyroid remnants and treatment of persistent or recurrent disease. It has been used for more than 8 decades and has increased the disease-free survival rate (1).

Radioactive iodine RAI (I-131) use ought to be goal-oriented by the American and European associations; the goals are categorized as treatment of unknown disease, adjuvant, and remnant ablation (2, 3). Remnant ablation improves the quality of further radioactive uptake and thyroglobulin levels; it is not intended to improve survival. In contrast, adjuvant therapy that removes microscopic foci after surgery is survival-oriented.

The majority of differentiated thyroid carcinomas can be managed by active surveillance or minimal surgery, and conventional intervention yielded no better outcomes (4, 5).

For appropriately selected patients with small, low-risk DTC, recent evidence shows that several minimally invasive and remote-access approaches, including trans-oral vestibular approach thyroidectomy, axillary/areolar endoscopic, and some robotic techniques) can provide comparable short-term oncologic outcomes and complication rates to conventional open thyroidectomy while offering improved cosmesis and patient satisfaction, but they require greater operative time, specialized training/volume, careful patient selection, and sometimes show lower lymph-node yield or higher cost (6–8).

RAI (I-131) is administered according to the risk stratification of thyroid cancer, and the dose is personalized and patient-specific. However, RAI (I-131) is costly, needs patient isolation, and is associated with long hospital stay, secondary malignancy, and women’s infertility (9–11). The effects of RAI (I-131) on women’s reproductive health and fertility (ovarian reserve) are a matter of controversy (12, 13). Ovarian reserve is a key determinant of fertility, influencing the chances of spontaneous conception. It predicts response to fertility treatments (e.g., IVF) and helps tailor stimulation protocols (14). Ovarian reserve reflects the quantity and quality of oocytes remaining within the ovaries at a given time and serves as a key determinant of female reproductive potential. Ovarian aging is characterized by a progressive depletion of the follicular pool accompanied by functional impairment of the remaining follicles. With advancing age, both follicular number and oocyte competence decline, leading to reduced fecundity and an increased risk of miscarriage. In clinical practice, diminished ovarian reserve is typically defined by biochemical thresholds, most commonly an anti-Müllerian hormone (AMH) level < 0.7 ng/mL and/or a follicle-stimulating hormone (FSH) level > 15 IU/L (15, 16). The AMH, which is the inhibitor of male reproductive structures during fetal development, is transforming growth factor beta (TGF-β) and is a marker of ovarian reserve in women (17). AMH plays an important role in female fertility and reproductive biology through the production of ovarian follicles (18). AMH plays an important role male sex differentiation by inducing the regression of the müllerian ducts, and it is the best marker of ovarian reserve. AMH is expressed by granulosa cells of growing follicles from the primary up to the small antral stage, then its expression disappears after the secretion of the secretion of follicle-stimulating hormone (FSH). However, some secretion continues in the cumulus cells of preovulatory follicles (19, 20). FSH was identified in the 1980s as a suppressor of gonadotropin-releasing hormone induced by luteinizing hormone (LH), with urinary/serum FSH testing and AMH levels are commonly used biomarkers to assess ovarian reserve and estimate a woman’s natural fertility potential (21, 22). This meta-analysis aimed to evaluate whether RAI therapy impairs ovarian reserve as assessed by anti-Müllerian hormone (AMH) and follicle-stimulating hormone (FSH) levels.

## Methods

### Study design

Systematic review; the review was not registered in PROSPERO registration because of the retrospective design of the included studies.

### Participants

Females who received RAI (I-131) for differentiated thyroid carcinoma.

### Eligibility criteria according to PICOS

**Figure 1:**
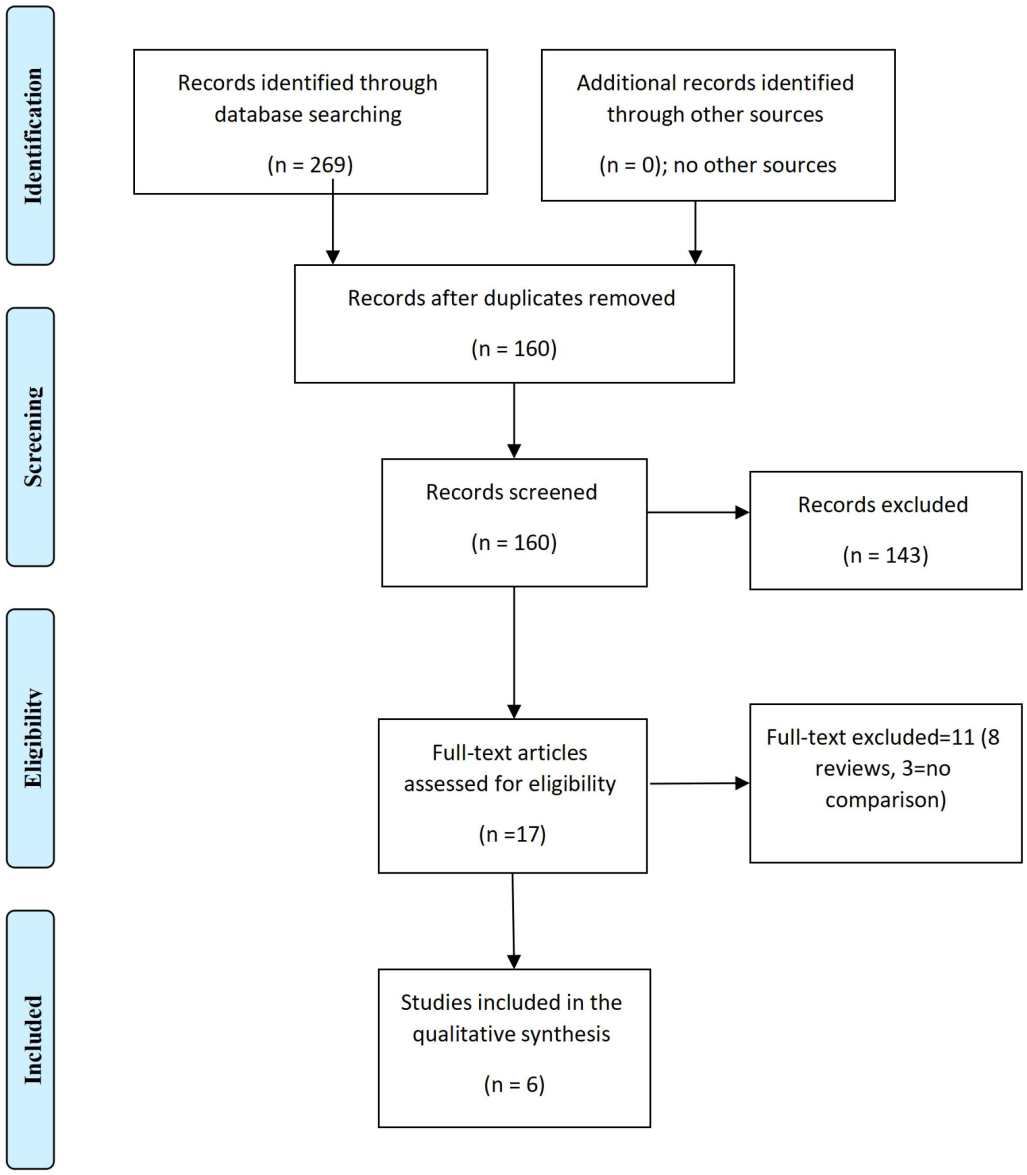
The effects of radioactive iodine RAI (I-131) (for differentiated thyroid carcinoma) on ovarian reserve (The PRISMA Chart).

This meta-analysis was conducted during October and November 2024 in accordance with the PRISMA Recommendation **Figure 1**.

### Inclusion criteria

We included cross-sectional studies, retrospective and prospective studies, and case-control studies assessing the effects of RAI (I-131) on ovarian reserve.

### Exclusion criteria

Experts’ opinions, editorials, case reports, and series were excluded from the study.

### Interventions

RAI (I-131), used for DTC.

### Outcome measures

The effects of RAI (I-131) on ovarian reserve (the levels of FSH and AMH before and after RAI (I-131) were reported.

### Comparators

Women who received radioactive iodine RAI (I-131) for differentiated thyroid carcinoma and controls.

### Systematic review protocol

Not done.

### Search strategy

The two authors independently searched PubMed, MEDLINE, Google Scholar, and EBSCO for relevant articles published in the English language. The literature search was conducted during October and November 2024, and publications published in the last ten years (from 2015 up to 2024) were included. The keywords used were ovarian reserve, ovarian function, female fertility, radioactive iodine RAI (I-131), follicle-stimulating hormone (FSH), and anti-Müllerian hormone (AMH), MeSH terms Iodine Radioisotopes, Ovarian Reserve, Thyroid Neoplasms. Two hundred and sixty-nine studies were retrieved, and one hundred and sixty remained after duplication removal; of them, 17 full texts were screened. However, only six studies were included in the final meta-analysis. Eight reviews and three texts without comparisons were excluded. The two authors (S. B and H. M blindly searched the literature, the excluded studies were discussed, and any discrepancy was solved by agreement.

### Data sources

PubMed, MEDLINE, Google Scholar, and EBSCO.

### Data extraction

A structured checklist was used to gather the author’s name, country, year of publication, and the levels of FSH and AMH before and after RAI (I-131) **Table 1**.

**Table 1:** The effects of Radioactive iodine RAI (I-131), on Anti-Müllerian hormone (AMH) and follicle-stimulating hormone (FSH).

Author	Country	Type of Study	Age/years cases	Age/years control	Follow-up	Anti-Müllerian hormone before ng/mL	Anti-Müllerian hormone after ng/mL	Number of patients	Number of controls
The effects of Radioactive iodine RAI (I-131), on Anti-Müllerian hormone (AMH)/mIU/mL									
Adamska et al., 2021 (23)	Poland	Prospective	33	33	12 months	2.7 ± 1.1	1.85 ± 1.45	25	25
Evranos et al., 2018 (24)	Turkey	Prospective	31.15 ± 4.83	31.15 ± 4.83	19months	8.87 ± 12.1	3.1 ± 3.01	33	33
Giusti et al., 2018 (25)	Italy	Prospective	40.7 ± 6.7 7.4	41.6 ±	7.1 ± 0.9 years	17.5 ± 4.7	10.7 ± 1.7	23	34
Hosseini et al., 2023 (26)	Iran	Prospective	29.53 ± 4.53	29.53 ± 4.53	12 months	2.25 ± 0.55	1.94 ± 0.58	60	60
Mittica et al., 2020 (27)	Italy	Prospective	41.2 ± 7.5	42.4 ± 9.2	24 months	8.71 ± 10.54	9.97 ± 11.46	30	59
Yaish et al., 2018 (28)	Israel	Prospective	34	34	12 months	3.25 ± 2.75	2.36 ± 1.88	24	24
The effects of Radioactive iodine RAI (I-131) on follicle-stimulating hormone (FSH)/mIU/ml									
Adamska et al., 2021 (23)	Poland	Prospective	33	33	12 months	5.7 ± 1.7	6.75 ± 1.85	25	25
Evranos et al., 2018 (24)	Turkey	Prospective	31.15 ± 4.83	31.15 ± 4.83	19months	9.64 ± 8.28	12.45 ± 7.52	33	33
Mittica et al., 2020 (27)	Italy	Prospective	41.2 ± 7.5	42.4 ± 9.2	24 months	15.3 ± 15.4	12.6 ± 12.4	30	59

### Statistical analysis

The RevMan version 5.4, Oxford, United Kingdom, was used to analyze the continuous data from six studies to assess the effects of radioactive iodine (I-131) on FSH and AMH. The data were assessed, and the mean difference 95% confidence interval was used for Forest Plots, and Funnel Plots were generated for heterogeneity*. I*^2^ was used to assess the heterogeneity among studies (*I*^2^ > 50% was considered high). The random effect was used for AMH due to the significant heterogeneity, and the fixed effect was used for the FSH arm because of non-significant heterogeneity. The chi-squared test and the weighted average effect size (Z) were calculated. A sub-analysis was used for the AMH outcome by excluding studies contributing most to heterogeneity (by assessing Heterogeneity Impact, and removing outliers with extreme effect size, then we checked if heterogeneity (I²) decreases and whether the pooled effect size changes. A P-value of < 0.05 was considered significant.

### Risk of bias assessment and quality of evidence

The Newcastle Ottawa Scale risk of bias assessment was used (29). All the included studies were of good quality. The GRADE Evidence was used to the assess the grade of evidence **Tables 2**, **3**.

**Table 2:** Newcastle Ottawa risk of bias of observational studies.

Author	Selection bias score	Compatibility score	Outcome bias score	Overall bias score	Quality
Adamska et al., 2021 (23)	4	2	3	9	Good
Evranos et al., 2018 (24)	4	2	3	9	Good
Giusti et al., 2018 (25)	4	2	3	9	Good
Hosseini et al., 2023 (26)	4	2	3	9	Good
Mittica et al., 2020 (27)	4	2	3	9	Good
Yaish et al., 2018 (28)	4	2	3	9	Good

12 months to.

**Table 3:** Analysis of the quality of evidence by Grading of Recommendations Assessment, Development, and Evaluation (GRADE).

Outcome	Studies	Study design	Risk of bias	Inconsistency	Indirectness	Imprecision	Other considerations	Certainty of evidence
AMH	6	Prospective=4, case-control=2	serious	Serious (*I^2^* = 90%)	Not serious	Not serious	None	Very low
FSH	3	Prospective=2, case-control=1	Serious	Not serious (*I^2^* = 7%)	Not serious	Not serious	None	Very low

## Results

### Characteristics of the included studies

There were six studies, all were prospective studies, the follow-up period ranged from 12 months to 7.1 ± 0.9 years, three studies were published in Europe, and three from Asia, and the total number of patients were 430.

### Findings

**Figure 2:**
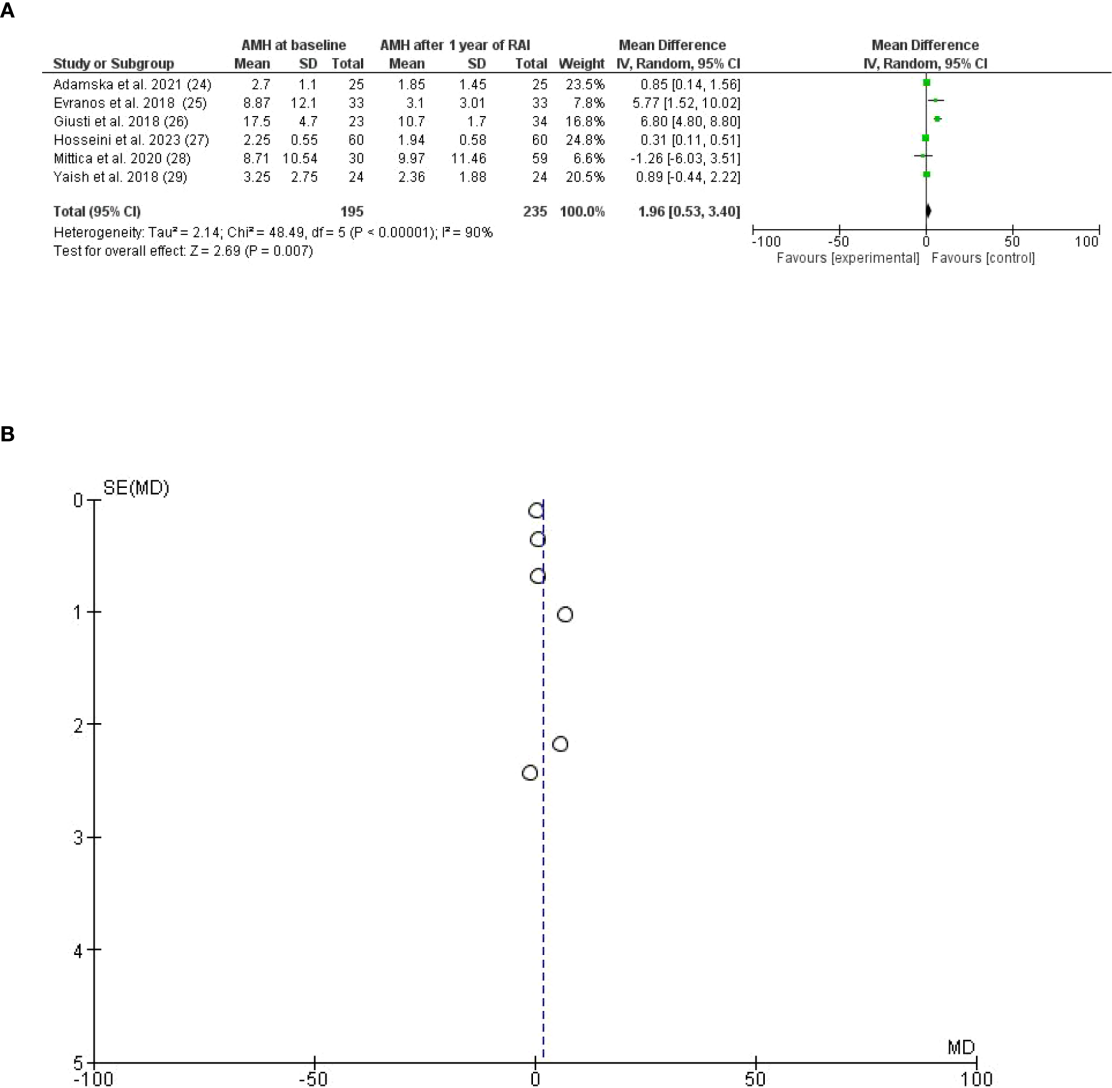
**(A)** (Forest Plot). Anti-Müllerian hormone (AMH) among patients receiving radioactive iodine RAI (I-131) for differentiated thyroid carcinoma after 1 year of radioactive iodine treatment. **(B)** Anti-Müllerian hormone (AMH) among patients receiving radioactive iodine RAI (I-131) for differentiated thyroid carcinoma after 1 year of radioactive iodine treatment (Funnel Plot).

In the present meta-analysis, six studies on Anti-Müllerian hormone (AMH) outcomes were included (23–28). AMH was significantly lower after RAI (I-131) with a mean difference (MD) of 1.96. 95% *CI*, 0.53 to3.40, P-value for overall effect, and Z = 2.69, 0.007. A significant heterogeneity was observed, *I*^2^ = 90%, the Chi-square=48.49, the standard difference was 5, and the P-value for heterogeneity was <0.001, **Figures 2A, B**.

**Figure 3:**
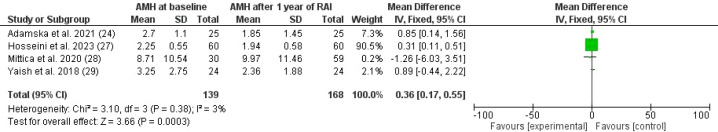
Anti-Müllerian hormone (AMH) among patients receiving radioactive iodine RAI (I-131) for differentiated thyroid carcinoma (after removing studies with high contribution to heterogeneity).

A sub-analysis was conducted after removing studies with high heterogeneity in which the Anti-Müllerian hormone (AMH) was lower in the RAI (I-131) therapy group compared to baseline and controls, MD, 0.36. 95% *CI*, 0.17 to 0.55, P-value for overall effect, 0.0003, and Z = 3.66, no significant heterogeneity was observed, *I*^2^, 3%, Chi-square, 3.10, the standard difference was 3, and the P-value for heterogeneity was 0.38 **Figure 3**.

**Figure 4:**
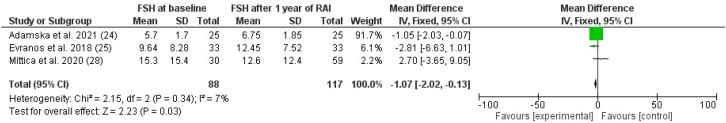
Follicle-stimulating hormone (FSH) in women receiving radioactive iodine RAI (I-131) for differentiated thyroid carcinoma.

Regarding follicle-stimulating hormone (FSH), only three studies were included (23, 24, 27) there was statistically significant increase after radioactive iodine RAI (I-131) (levels 30.64 versus 31.8), MD -1.07, 95% *CI*, -2.02 to-0.13, P-value for overall effect, 0.03, and Z = 2.23. No heterogeneity was observed, *I*^2^ = 7.0%, Chi-square, 2.15, the standard difference was 2, and the P-value for heterogeneity was 0.34 **Figure 4**.

## Discussion

In this meta-analysis, AMH was significantly lower after RAI (I-131) with a mean difference (MD) of 1.96. 95% *CI*, 0.53 to3.40. AMH were lower after removing studies with significant heterogeneity, MD, 0.36. 95% *CI*, 0.17 to 0.55. While FSH levels were significantly higher after radioactive iodine RAI (I-131) (levels 30.64 versus 31.8), MD -1.07, 95% *CI*, -2.02 to-0.13.

The current findings are in line with a meta-analysis that included only four studies and found a 1.50 ng/mL reduction of Anti-Müllerian hormone (AMH) (10). Another meta-analysis included the same number of studies (four) and reported a decline in Anti-Müllerian hormone (AMH) at three months following a single dose of radioactive iodine RAI (I-131). In addition, the low level persisted for one year. Anagnostis et al. (13) investigated the effects of radioactive iodine RAI (I-131) on follicle-stimulating hormone (FSH) and found no significant effects at three, six months, and one year, their findings were contradicting the current findings in which FSH levels were significantly higher after RAI (I-131). Importantly, Anagnostis and colleagues included only data from two studies post-radioactive iodine RAI (I-131). In addition, there was a substantial heterogeneity (96.8%).

Wu et al. (30) conducted a large retrospective study that found a decreased birth rate among women who received RAI (I-131), and the effect was greater among the older age group. Similarly, previous studies reported metrorrhagia, oligomenorrhea, and amenorrhea in 20-30% of women receiving radioactive iodine RAI (I-131). However, the changes were temporary and usually did not exceed one year (31–33).

Another piece of evidence of the negative impact of RAI (I-131) on ovarian reserve is the findings of Ceccarelli et al. (34), who observed a decreased fertility period among women receiving this modality of therapy. The advancement in menopause observed by the authors implies a negative effect on ovarian reserve. Given the current findings, a shared decision with women who are planning to receive radioactive iodine RAI (I-131) is of paramount significance, and counseling regarding reproductive health is vital.

Radioactive iodine RAI (I-131) is widely used in differentiated thyroid carcinoma at different doses (35). However, RAI (I-131) is not without side effects; the side effects range from salivary gland pain, xerostomia, and sialadenitis to secondary malignancies and ovarian failure (36). The important concern regarding RAI (I-131) use is its effects on ovarian reserve (AMH and FSH).

The effects of RAI (I-131) on AMH and FSH are due to its gonadotoxic effects. The primary mechanism involves radiation-induced oxidative stress and direct DNA damage to ovarian follicles, leading to apoptosis of granulosa cells and depletion of the primordial follicle pool. As ovarian follicular function declines, negative feedback inhibition on the hypothalamic-pituitary axis is reduced, leading to compensatory elevations in follicle-stimulating hormone (FSH) levels (7, 12).

Anti-Müllerian hormone (AMH) is the most sensitive indicator of ovarian reserve due to its intracycle and intercycle variability. AMH is sensitized in granulosa cells of the growing ovarian follicles, and decreases progressively with increasing age, and shows the lowest level before menopause (36).

Ovarian reserve studies are an important option for women who have not yet fulfilled their reproductive goals before receiving radioactive iodine RAI (I-131) therapy (37). The American Society of Clinical Oncology (ASCO) recommends offering oocyte cryopreservation for fertility preservation in women at risk of diminished ovarian reserve (38, 39). Therefore, it is advisable that women scheduled for RAI (I-131), particularly those of reproductive age, undergo baseline assessment of ovarian reserve markers such as anti-Müllerian hormone (AMH) and follicle-stimulating hormone (FSH). If results suggest reduced reserve, fertility preservation measures (including oocyte or embryo cryopreservation) should be discussed before therapy.

A multidisciplinary approach involving endocrinologists, oncologists, reproductive specialists, and surgeons is essential for individualized decision-making. Proper patient selection is critical, especially among those with low to intermediate risk differentiated thyroid carcinoma (DTC), where the benefit of RAI (I-131) in preventing recurrence remains controversial (3, 40, 41). Given that RAI (I-131) distributes systemically during both uptake and elimination, it can cause various complications such as xerostomia, dysgeusia, sialadenitis, gonadal dysfunction, and, rarely, secondary malignancies and infertility (42–44).

Hence, for women of childbearing age, shared decision-making should include counseling on the potential impact of RAI (I-131) on fertility, consideration of delaying therapy until after fertility preservation when feasible, use of the lowest effective RAI (I-131) dose, and post-treatment monitoring of ovarian function. Such individualized care ensures that the therapeutic benefits are balanced against reproductive and long-term risks.

The strength of our study is that we included the largest number of studies, included the most recent updates, and found no effect of radioactive iodine RAI (I-131) on follicle-stimulating hormone (FSH), supporting the previous meta-analysis.

### Study limitations

The study limitations were the small number of included studies, the difference in the follow-up period in the included studies, variable Anti-Müllerian hormone (AMH) assays, lack of long-term follow-up, and the significant heterogeneity observed. The source of heterogeneity observed in this study could be due to differences in RAI (I-131) dose, age of the patients, timing, and duration of the included studies.

## Conclusion

Although RAI (I-131) therapy appears to transiently reduce Anti-Müllerian hormone (AMH) levels, the clinical impact on fertility remains uncertain. Follicle-stimulating hormone (FSH) were higher after RAI (I-131). Larger prospective studies using standardized Anti-Müllerian hormone (AMH) assays and dose stratification are required to confirm these findings.

## Data Availability

The datasets presented in this study can be found in online repositories. The names of the repository/repositories and accession number(s) can be found in the article/supplementary material.
